# Rapid and highly sensitive immunoassay using an ultra-thin immuno-wall microfluidic device with a sequential fluorescence signal increment method

**DOI:** 10.1007/s00216-025-05916-x

**Published:** 2025-05-28

**Authors:** Xiang Zhou, Toshihiro Kasama, Ryo Miyake

**Affiliations:** Department of Bioengineering, Graduate School of Engineering, The University of Tokyo, Tokyo, Japan

**Keywords:** SARS-CoV-2 virus, High sensitivity, Fluorescence immunoassay, Microfluidic device, Photopolymer

## Abstract

We present a rapid and highly sensitive immunoassay platform based on an ultra-thin immuno-wall microfluidic device with an easy-to-perform sequential fluorescence signal increment method. The ultra-thin immuno-wall was fabricated using a special type of water-soluble photopolymer mixed with streptavidin via photolithography. During photolithography, the photopolymer formed a three-dimensional cross-linked structure, and streptavidin was immobilized in the cross-linked structure based on the click chemistry reaction. The immobilized streptavidin was used to immobilize biotin-conjugated antibodies on the cross-linked structure to capture biomarkers, forming immune complexes on the surface, known as an “immuno-wall.” A sequential fluorescence signal increment method utilizes two different fluorescence-labeled antibodies with high affinity that were incubated several cycles in the immuno-wall to enhance the fluorescence signal. Moreover, an ultra-thin immuno-wall was developed to reduce the nonspecific binding and increase the signal-to-noise ratio. To evaluate the performance of this immunoassay platform, the spike protein from the SARS-CoV-2 virus was selected as the target biomarker. This immunoassay platform exhibited a limit of detection of 0.01 ng/mL, and the detection time was 30 min, which is comparable to rapid antigen tests. This immunoassay platform demonstrates significant potential for early-phase disease diagnosis.

**Supplementary Information:**

The online version contains supplementary material available at 10.1007/s00216-025-05916-x.

## Introduction

Rapid and sensitive immunoassay techniques have become increasingly crucial in modern healthcare, particularly in the fields of early disease diagnosis and precision medicine [1–3]. Traditional immunoassay techniques often face a fundamental trade-off between detection sensitivity and speed. Well-established techniques such as enzyme-linked immunosorbent assay (ELISA) provide relatively high sensitivity and high reproducibility but require lengthy processing times [4, 5], whereas other rapid test techniques such as lateral flow immunoassay (LFIA) offer sample-in-result-out detection but frequently sacrifice detection accuracy [6, 7]. These limitations become particularly challenging when dealing with time-sensitive diagnoses in early disease phases with a minimal biomarker load [8, 9].

The need for rapid and highly sensitive immunoassays has become particularly apparent during the COVID-19 pandemic [10–12]. The COVID-19 pandemic, caused by the novel coronavirus, SARS-CoV-2, in which asymptomatic cases can be infectious, has become a global public health crisis since 2020 [13–15]. Hence, rapid and sensitive detection, timely diagnosis, effective treatment, and real-time monitoring for asymptomatic or presymptomatic individuals are crucial for controlling the transmission of infection during early phases [16–19]. Currently, the main immunoassay application authorized by the US Food and Drug Administration for COVID-19 is the rapid antigen test based on LFIA. It provides a rapid result in 20–30 min but has a relatively high false negative rate, particularly for asymptomatic cases with low viral loads [11, 20, 21]. Consequently, the results of the rapid antigen test can only be used as a reference for screening, and suspicious patients still need to undergo PCR tests to obtain accurate diagnostic results, which are time-consuming (24–48 h), and the specialized lab-based equipment precludes its implementation for time-sensitive screening and testing [10, 22–24]. To date, there are still few immunoassay platforms addressing the need for rapid and highly sensitive diagnosis.

**Fig. 1:**
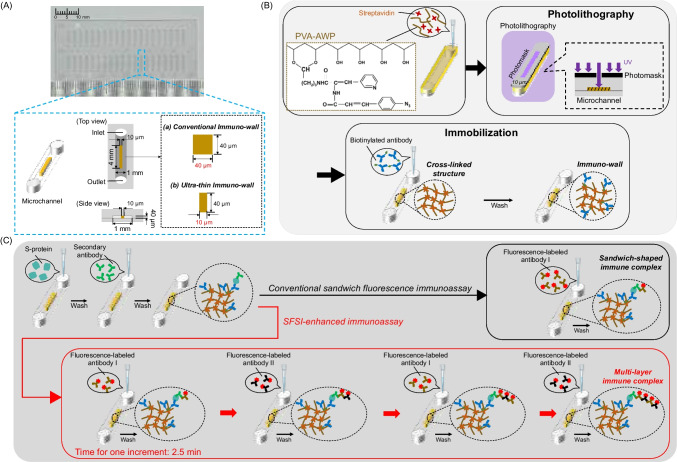
**A** Photograph of a piece of a COP chip with microchannels and a schematic of one microchannel with the size of an immuno-wall. **B** Schemes for the fabrication processes for an immuno-wall. **C** Schemes for sandwich fluorescence immunoassay and SFSI-enhanced immunoassay

In our previous study, we reported a rapid and sensitive immunoassay technique based on a microfluidic immuno-wall device (defined here as a conventional immuno-wall with a width of 40 μm) (Fig. 1A) [25–28]. In the microchannel, a photopolymer-based, wall-shaped, three-dimensional structure was fabricated and used to immobilize biomarkers. A sandwich fluorescence immunoassay was conducted on the immuno-wall. For this immuno-wall, the detection time was 20 min, and the sensitivity was 10 ng/mL. However, to meet the clinical sensitivity needed for COVID-19 detection in the early phases of infection, further improvement in sensitivity is necessary [29–31].

The strategies to improve the sensitivity of immunoassays using immuno-wall are usually categorized into two approaches. One is to enhance the fluorescence signal, and the other is to reduce the background noise. In this study, we applied both by developing an easy-to-perform sequential fluorescence signal increment (SFSI) method to enhance the fluorescence signal, along with a low-nonspecific-binding ultra-thin immuno-wall to reduce the background noise, thereby improving the sensitivity. The SARS-CoV-2 spike protein (S-protein) was selected as the target biomarker [32, 33]. Given that the S-protein is a surface protein of the SARS-CoV-2 virus, there is no need for extraction, significantly shortening the operation time of the immunoassay. The load of the S-protein in early infection phases is approximately in a range of 0.01–0.1 ng/mL [29, 31, 34, 35]. In this study, we aim to enhance the sensitivity of our immunoassay platform to match the minimum level of this range (0.01 ng/mL), while also targeting a detection time comparable to rapid antigen tests, within 30 min.

## Materials and methods

### Reagents and materials

Phosphate-buffered saline (PBS, pH 7.4) was purchased from Thermo Fisher Scientific, Inc. (Waltham, MA). Blocker-bovine serum albumin (BSA) was purchased from Pierce (Rockford, IL). Tween-20 was purchased from Sigma-Aldrich Co., LLC (P9416; St. Louis, MO). Recombinant streptavidin was purchased from ProSpec-Tany Technogene, Ltd. (East Brunswick, NJ). Polyvinyl alcohol azide-unit pendant water-soluble photopolymer (PVA-AWP, 6% aq) was purchased from Toyo Gosei Co., Ltd. (Tokyo, Japan). Cyclic olefin copolymer (COP) microchips with 40 microchannels were purchased from Sumitomo Bakelite Co., Ltd. (Tokyo, Japan). PBS containing 0.5% Tween-20 and 0.5% BSA was used as the washing buffer for fabrication and immunoassay. The 1% BSA was diluted to 0.5% BSA by using PBS (0.5% BSA/PBS buffer) and was used as the solvent for biomarkers. Biotinylated anti-SARS-CoV-2 monoclonal human IgG antibody was purchased from Novus Biologicals, LLC (CO, USA). SARS-CoV-2 spike (trimeric) recombinant protein (8×His-Tag) was purchased from Cell Signaling Technology, Inc. (Danvers, MA). Anti-SARS-CoV-2 spike S2 rabbit IgG antibody was purchased from Sino Biological, Inc. (Suzhou, China). Dylight 650-conjugated goat anti-rabbit polyclonal IgG antibody (fluorescence-labeled antibody I), Dylight 650-conjugated rabbit anti-goat polyclonal IgG antibody (fluorescence-labeled antibody II), and Dylight 650-conjugated rabbit anti-human polyclonal IgG antibody were purchased from Abcam Inc. (MA, USA). Saliva swab was purchased from SalivaBio Oral Swab (SOS) (Salimetrics, LLC., USA).

### Design and fabrication of immuno-wall in microchannels

The immuno-wall, which is used for the immobilization of antibodies, was fabricated by utilizing the special optical and chemical properties of PVA-AWP. PVA-AWP is a water-soluble photopolymer consisting of photoreactive azide functional groups bound to the main chain of PVA, as shown in Fig. 1B. Irradiating the azide functional groups with UV light with a wavelength range of 315–325 nm initiates cross-linking. Simultaneously, the azide functional groups (-N_3_) react with the amino functional groups (-NH_2_) on the biomolecules to form chemical bonds via the click chemistry reaction, immobilizing them on the cross-linked structure, thereby forming the immuno-wall.

The fabrication process is illustrated in Fig. 1B. First, a photomask was aligned with the COP microchip, and both were fixed into a order-made holder (Fig. S1). The sizes of the microchannel and immuno-wall are shown in Fig. 1A. Second, a microchannel was filled with a mixture (0.6 μL, volume ratio 1:1) of PVA-AWP and 10 mg/mL streptavidin dissolved in PBS using a high-viscosity pipette (PIPETMAN; Gilson S.A.S.; Villiers le Bel, France). Third, photolithography was performed for 10 s using a UV light (325 nm, 20 mW/cm^2^) generated from a mercury lamp (LA-410UV; Hayashi Watch Works; Tokyo, Japan) to pattern the three-dimensional wall-shaped structure. After photo-fabrication, the uncured mixture of PVA-AWP and streptavidin was washed out, followed by washing with the washing buffer 10 times. Then, the microchannel was filled with washing buffer to prevent it from drying out and nonspecific binding. The structure of the immuno-wall extended from the floor to the roof of the microchannel so that only the lateral side of the immuno-wall would be in contact with the loaded sample during the immunoassay. Finally, biotinylated anti-SARS-CoV-2 S-protein monoclonal human IgG antibody (immobilized antibody) (0.6 μL, 50 μg/mL) in 0.5% BSA/PBS buffer was injected into the microchannel containing the streptavidin-modified immuno-wall and incubated for 1 h at room temperature (25 ℃). After incubation, the excess antibody was removed by using a vacuum pump. Then, the washing buffer was added to the microchannel and allowed to penetrate the immuno-wall for 1 min, followed by washing five times with the washing buffer. After washing, the microchannel was filled with washing buffer to prevent nonspecific binding, and the inlet and outlet of the microchannel were sealed with tape to prevent dryness.

### Sandwich fluorescence immunoassay

The procedures of sandwich fluorescence immunoassay are illustrated in Fig. 1C. First, the SARS-CoV-2 spike protein (S-protein) in 0.5% BSA/PBS buffer (0.6 μL) was introduced into the microchannel and allowed to incubate for 15 min at room temperature (25 ℃). After incubation, the excess sample was removed using a vacuum pump, and the microchannel was then washed. Second, anti-SARS-CoV-2 spike S2 rabbit IgG antibody (secondary antibody) (50 μg/mL) in 0.5% BSA/PBS buffer (0.6 μL) was introduced into the microchannel and allowed to incubate for 1 min. After incubation, the excess sample was removed using a vacuum pump, and then the microchannel was washed. Next, fluorescence-labeled antibody I (F-I) (0.6 μL, 50 μg/mL) in 0.5% BSA/PBS buffer was introduced into the microchannel and allowed to incubate for 30 s. Then, the excess sample was removed using a vacuum pump, and the microchannel was washed. Finally, this process afforded the sandwich-shaped immune complexes with the immobilized antibody-S-protein-secondary antibody-F-I on the lateral side of the immuno-wall. Fluorescence images of the lateral side of the immuno-wall were obtained using fluorescence microscopy. The washing process for each step of the assay was the same: the microchannel was filled with the washing buffer, and the washing buffer was kept in the channel for 1 min to ensure it penetrated the immuno-wall sufficiently. Finally, the microchannel was washed five times with the washing buffer.

### SFSI-enhanced immunoassay

**Fig. 2:**
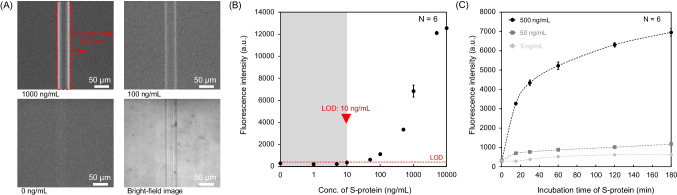
**A** The images from the top left to the bottom right are fluorescence images of the immuno-wall with S-protein concentrations of 1000, 100, and 0 ng/mL, followed by a bright-field image. **B** Calibration curve for S-protein detection in 0.5% BSA/PBS buffer. **C** Plot of fluorescence intensity vs. incubation time for S-protein concentrations of 500, 50, and 5 ng/mL. For **B** and **C**, the fluorescence intensities were plotted as mean ± SD (*N* = 6), and the dotted line represents the LOD, which is 3 times the SDs above the negative control (0 ng/mL)

The SFSI-enhanced immunoassay was designed by co-incubating two different fluorescence-labeled antibodies with high affinities on the sandwich-shaped immune complex to enhance the fluorescence signal directly, particularly for low-concentration samples, as illustrated in Fig. 2C. After the incubation of the secondary antibody, F-I (0.6 μL, 50 μg/mL) was introduced into the microchannel and incubated for 30 s, followed by the incubation of fluorescence-labeled antibody II (F-II) (0.6 μL, 50 μg/mL) in an alternating sequence of F-I → F-II → F-I → F-II, and so forth. After each incubation cycle, the excess sample was removed by using a vacuum pump, and then the microchannel was washed. The operation time of each cycle, including the washing process, was approximately 2.5 min. For example, four cycles took about 10 min. Finally, the multi-layer immune complexes were formed on the lateral side of the immuno-wall, and the fluorescence images of the lateral side of the immuno-wall were acquired using fluorescence microscopy.

### Determination of fluorescence intensity

The fluorescence images of the immuno-wall were acquired using a fluorescence microscope (GX71; Olympus Corp., Tokyo, Japan) equipped with a CCD camera (EM-CCD c9100; Hamamatsu Photonics, Shizuoka, Japan). A Cy5 fluorescence filter (67-010-OLY; Edmund Optics, Inc., Barrington, NJ) was used to detect the Dylight 650 fluorophore conjugation. The exposure time for each image was 3 s. For each immuno-wall, three fluorescence images (top, middle, and bottom regions) were taken, and the mean value of the fluorescence intensities was calculated and used as the final fluorescence intensity for the corresponding sample.

Two methods were used to analyze the fluorescence intensity based on different detection areas. For the conventional immuno-wall, the entire immuno-wall area was selected as the detection area with the same number of pixels, as shown in Fig. 2A. For the ultra-thin immuno-wall with the SFSI-enhanced immunoassay, another method to determine the detection area was approached, where the lateral sides of the immuno-wall were selected as the detection area with the same number of pixels (8 pixels in width and 1000 pixels in length). This is because the contrast of the fluorescence signal between the middle of the immuno-wall (reduced background noise) and the lateral sides became relatively high, making it possible and advantageous to analyze only the lateral sides of the immuno-wall to obtain a higher signal-to-noise (S/N) ratio, as shown in Fig. S5. For both methods, the fluorescence intensities of the samples were calculated as the mean values of all pixels within the selected detection area, as shown in Fig. S5. The image analysis, including area selection and mean value calculation, was conducted automatically using a Python algorithm to ensure the selected area of the images contained the same amount of pixels. The fluorescence intensity values were normalized by subtracting the autofluorescence of the immuno-wall, which was used as the background. The limit of detection (LOD) was determined by using the signal at 3 standard deviations (SDs) above the negative control (0 ng/mL), which was employed in previous studies [25, 26, 28]

## Results and discussion

### Feasibility of conventional sandwich fluorescence immunoassay for S-protein

To evaluate the feasibility of the sandwich-shaped immune complex to detect the S-protein, the immunoassay was conducted using a conventional immuno-wall, following the immunoassay procedures mentioned above. The S-protein, secondary antibody, and F-I were incubated in the microchannel to form sandwich-shaped immune complexes. A calibration curve for S-protein detection is shown in Fig. 2B. It was confirmed that the fluorescence intensity increased with an increase in the concentration of the S-protein. The LOD was 10 ng/mL, and the detection range was 10–5000 ng/mL, with a total assay time of about 20 min. However, this sensitivity is insufficient for the target sensitivity, as mentioned in the “Introduction section.”

Since diffusion is the only factor affecting the contact efficiency between the antibody and antigen in the microchannel, which affects the sensitivity, we investigated the correlation between the incubation time of the S-protein and the sensitivity. In this experiment, 500, 50, and 5 ng/mL of S-protein were selected based on the calibration curve in Fig. 2B. The incubation times were 15, 30, 60, 120, and 180 min. A plot of the fluorescence intensity vs. the incubation time is shown in Fig. 2C. The fluorescence intensity did not increase significantly when the S-protein concentration was below 50 ng/mL, regardless of the incubation time. In other words, the correlation between the incubation time and the sensitivity decreases with a decrease in the S-protein concentration. Thus, when the concentration of the S-protein is low (especially less than LOD of 10 ng/mL), some other approaches are necessary to enhance the fluorescence signal. Hence, we designed the SFSI method to enhance the fluorescence signal of sandwich-shaped immune complexes formed on the immuno-wall. We assumed that the fluorescence signal of low-concentration samples would progressively increase with the increase of the incubation of fluorescence-labeled antibodies.

### Feasibility of SFSI-enhanced immunoassay using conventional immuno-wall

**Fig. 3:**
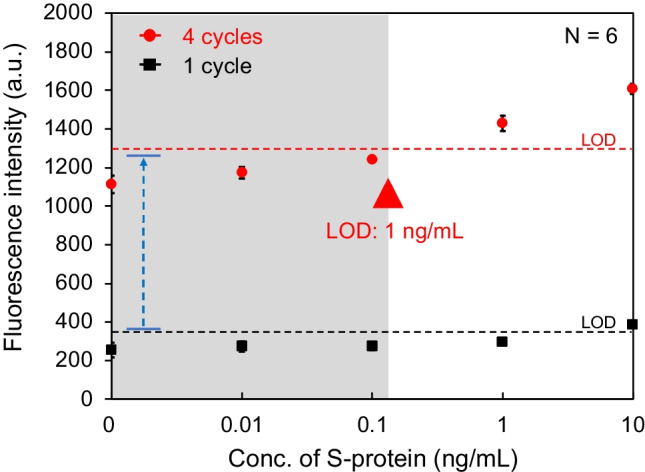
Calibration curves for S-protein detection in 0.5% BSA/PBS buffer with 1-cycle and 4-cycle enhancement using the conventional immuno-wall. The fluorescence intensities were plotted as mean ± SD (*N* = 6), and the dotted line represents the LOD, which is 3 times the SDs above the negative control (0 ng/mL)

To evaluate the SFSI-enhanced immunoassay towards rapid detection, the total incubation time of fluorescence-labeled antibodies (F-I and F-II) should be as short as possible. Thus, the total incubation cycles were set to four (two cycles for F-I and two cycles for F-II) so that all the immunoassay operations could be completed within 30 min. Before the evaluation of the SFSI-enhanced immunoassay, we designed and conducted an experiment to confirm the formation of multi-layer immune complexes. In this experiment, the S-protein at 100 ng/mL was used. After the formation of the sandwich-shaped immune complex (1 cycle), F-I and F-II (0.6 μL, 50 μg/mL) were incubated for 30 s, respectively, followed by washing. The fluorescence intensity of each immuno-wall was acquired and plotted in Fig. S2. The results demonstrated that the fluorescence signal did not increase after the additional incubation with F-I but increased after the incubation with F-II. This confirms that the concentration of F-I had reached saturation to bind with the secondary antibody to form the sandwich-shaped immune complex during the first cycle, and that multi-layer immune complexes formed as expected. Next, to confirm the feasibility of the SFSI-enhanced immunoassay, 0, 0.01, 0.1, 1, and 10 ng/mL of S-protein in 0.5% BSA/PBS buffer, which is below the LOD obtained from the calibration curve in Fig. 2B, were used, following the immunoassay procedures mentioned above. The calibration curves for four incubation cycles were plotted in Fig. S3, and the calibration curves for the 1-cycle (sandwich fluorescence immunoassay) and 4-cycle enhancement were plotted in Fig. 3. The plots showed that the fluorescence intensity increases as the incubation cycle of the fluorescence-labeled antibodies increases. The LOD was improved to 1 ng/mL with an assay time of 30 min. However, with the increase in the incubation cycle, the fluorescence intensity of the negative control (0 ng/mL) increased gradually as well. Thus, although the sensitivity was improved 10-fold, it did not reach the target sensitivity mentioned in the “Introduction section.”

Since we used a monoclonal antibody as the immobilized antibody and all antibodies forming immune complexes were selected from different clones to avoid cross-reactivity, cross-reactivity can be neglected. Hence, we assumed that the increasing fluorescence signal of the negative control is caused by the nonspecific binding with unreacted antibodies/antigens absorbed into the cross-linked structure of the immuno-wall, despite washing. To confirm this, in the next section, we investigated the nonspecific binding inside the immuno-wall.

### Nonspecific binding inside the immuno-wall and optimization of the structural properties of the immuno-wall

**Fig. 4:**
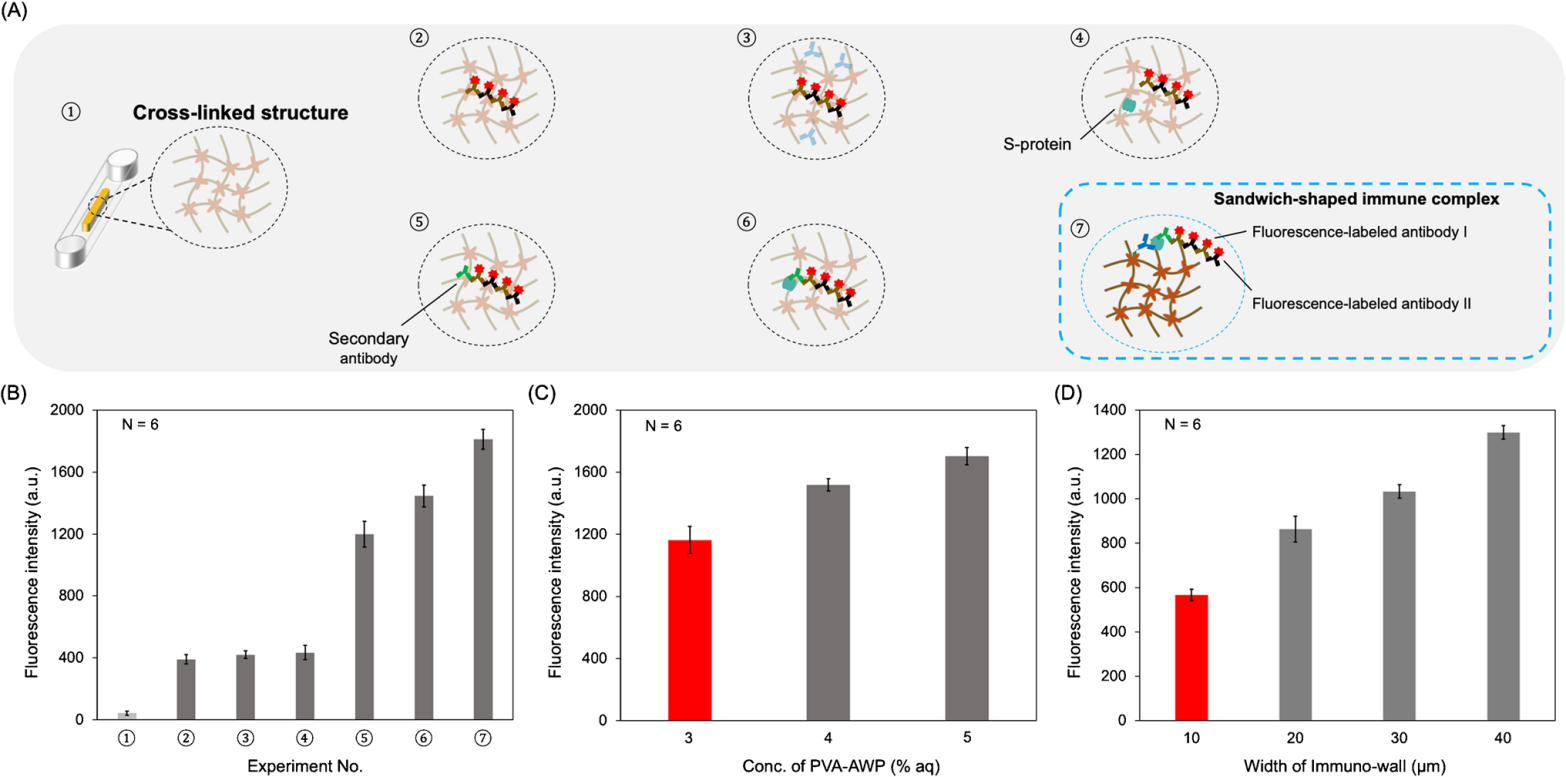
**A** Schematic of the combinations of antibodies/antigens from multi-layer immune complexes corresponding to Table 1. **B** Bar graph of fluorescence intensity vs. experiment number. **C** Bar graph of fluorescence intensity vs. concentration of PVA-AWP. **D** Bar graph of fluorescence intensity vs. width of Immuno-wall

To investigate the nonspecific binding of unreacted antibodies/antigens inside the immuno-wall, several combinations of antibodies/antigens were designed, as shown in Table 1 and Fig. 4A. The “+” means one incubation cycle, “++” means two, and “–” means no incubation. In this experiment, 50 μg/mL of the immobilized antibody, 50 μg/mL of the secondary antibody, 10 ng/mL of the S-protein, and 50 μg/mL of F-I and F-II were used during incubation. A bar graph corresponding to Table 1 is shown in Fig. 4B. A comparison of the results from ②–④, ⑤, and ⑥ indicated that there were unreacted antibodies/antigens in the cross-linked structure of the immuno-wall after washing. These unreacted antibodies/antigens increased the fluorescence signal after several incubation cycles of the fluorescence-labeled antibodies.

**Table 1:** Antibody/antigen combinations from the multi-layer immune complex

Experiment no.	①	②	③	④	⑤	⑥	⑦
Immobilized antibody	-	-	+	-	-	-	+
S-protein	-	-	-	+	-	+	+
Secondary antibody	-	-	-	-	+	+	+
F-I	-	++	++	++	++	++	++
F-II	-	++	++	++	++	++	++

To achieve a higher S/N ratio while maintaining a rapid operation time, instead of increasing the time used for washing, (a) the density of the cross-linked structure and (b) the width of the cross-linked structure were investigated and optimized based on the obstruction model as shown below:

$$D= {D}_{0}\times {(1-k\nu )}^\frac{3}{2}$$

where *D* is the diffusion coefficient, $\nu$ is the cross-linking density, and *k* is a constant dependent on the polymer-solvent system.

According to the obstruction model, a lower density and a smaller width of the cross-linked structure should allow the washing buffer to penetrate inside more efficiently to wash out the unreacted antibodies/antigens, etc. Thus, we investigated the influence of these two factors on the washing efficiency for nonspecific binding and optimized these two factors of the immuno-wall for SFSI-enhanced immunoassay.

#### (a) Optimization of the density of cross-linked structure

The photoreactive azide functional groups (-N_3_) (Fig. 1B) on PVA-AWP play a dominant role during the polymerization to form the cross-linked structure via photolithography. This means that the cross-linking density is correlated to the concentration of PVA-AWP. To confirm the effects of the cross-linking density on nonspecific binding, the fluorescence signal of the negative control (0 ng/mL) was studied using immuno-walls fabricated with different concentrations of PVA-AWP. In this experiment, the original PVA-AWP (6% aq) was diluted with a streptavidin solution to give solutions with concentrations of 1%, 2%, 3%, 4%, and 5% aq. To eliminate the influence of streptavidin, the final concentration of streptavidin in all samples was adjusted to 2 mg/mL. The immuno-walls with different concentrations of PVA-AWP were fabricated following the fabrication procedures mentioned above. However, immuno-walls with 1% and 2% aq PVA-AWP could not be fabricated. It is because the concentration of the photoreactive group was too low that the three-dimensional structure inside the microchannel was unstable; hence, the frictional force from the cross-linked structure on the top and bottom of the microchannel was too small, meaning that the cross-linked structure was destroyed during the washing process. After the fabrication of the immuno-wall, 0.5% BSA/PBS buffer (as negative control (0 ng/mL)), secondary antibody, F-I (2 cycles), and F-II (2 cycles) were incubated in the microchannel, and then the microchannel was washed following the washing process. If washing does not remove the unreacted secondary antibodies inside the immuno-wall, the fluorescence-labeled antibodies will react with them, causing an increase in the fluorescence signal. A plot of the fluorescence intensity vs. concentration of PVA-AWP is shown in Fig. 4C. It was confirmed that a lower cross-linking density exhibits a better washing efficiency, resulting in a weaker fluorescence signal. The immobilization ability of 3%, 4%, and 5% aq PVA-AWP was also confirmed, as shown in Fig. S4. Thus, 3% aq PVA-AWP was determined to be the optimal concentration for fabricating immuno-walls with the lowest cross-linking density and the highest S/N ratio for SFSI-enhanced immunoassay.

#### (b) Optimization of the width of immuno-wall

To determine the effects of the immuno-wall width on nonspecific binding, the fluorescence signals of negative controls (0 ng/mL) using immuno-walls with widths of 10, 20, 30, and 40 μm were studied. Widths less than 10 μm could not be fabricated because the three-dimensional structure inside the microchannel was unstable due to the frictional force from the cross-linked structure on the top and bottom of the microchannel being too small, meaning that the cross-linked structure was destroyed during the washing process. After the fabrication of the immuno-wall, 0.5% BSA/PBS buffer (as negative control (0 ng/mL)), secondary antibody, F-I (2 cycles), and F-II (2 cycles) were incubated in the microchannel, and then the microchannel was washed following the washing process. As mentioned above, if unreacted secondary antibodies remain inside the immuno-wall after washing, the fluorescence-labeled antibodies will react with them, causing an increase in the fluorescence signal. A plot of the fluorescence intensity vs. the width of the immuno-wall is shown in Fig. 4D. It was confirmed that the washing efficiency is better for a thinner immuno-wall, resulting in a weaker fluorescence signal. An immuno-wall width of 10 μm was determined to be optimal for the highest S/N ratio for SFSI-enhanced immunoassay. The 10 μm immuno-wall was defined as an “ultra-thin immuno-wall.”

### Performance check of SFSI-enhanced immunoassay using ultra-thin immuno-wall

**Fig. 5:**
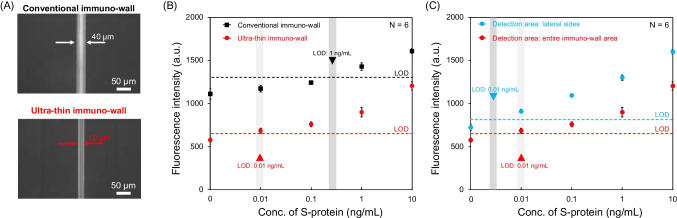
**A** Fluorescence images for a conventional immuno-wall and an ultra-thin immuno-wall after SFSI-enhanced immunoassay (S-protein 10 ng/mL). **B** Calibration curves for S-protein in 0.5% BSA/PBS buffer using conventional and ultra-thin immuno-walls with the detection area of the entire immuno-wall area. **C** Comparison of the calibration curves for the fluorescence intensity vs. the S-protein concentration in 0.5% BSA/PBS buffer using an ultra-thin immuno-wall with the detection area of the entire immuno-wall area vs. lateral-side area. For **B** and **C**, the fluorescence intensities were plotted as mean ± SD (*N* = 6). The dotted line represents the LOD, which is 3 times the SDs above the background (BG) (0 ng/mL)

So far, we have investigated two fundamental properties related to nonspecific binding and optimized the immuno-wall for SFSI-enhanced immunoassay. As defined above, an ultra-thin immuno-wall is a three-dimensional structure with 3% aq PVA-AWP and a width of 10 μm. To evaluate the performance of SFSI-immunoassay using an ultra-thin immuno-wall, 0, 0.01, 0.1, 1, and 10 ng/mL of S-protein in 0.5% BSA/PBS buffer were used. The calibration curve for the fluorescence intensity vs. S-protein concentration is shown in Fig. 5B. The LOD was improved to 0.01 ng/mL, which is 1000-fold over the sandwich fluorescence immunoassay using a conventional immuno-wall, due to the enhanced signal and the reduced background noise.

Furthermore, when we analyzed the profile of the fluorescence signal of the immuno-wall, we observed that, since the background noise of the ultra-thin immuno-wall was significantly reduced, the contrast between the fluorescence signal on the lateral sides and the interior of the immuno-wall was enhanced (Fig. 5A and Fig. S5). Thus, we attempted to apply a new method to analyze the fluorescence intensity by selecting both lateral sides as the detection area, rather than the entire area of the immuno-wall. Calibration curves for the SFSI-enhanced immunoassay using the ultra-thin immuno-wall with different detection areas were plotted in blue and red in Fig. 5C, and the details of the calculations are shown in Fig. S5. For both ways, the LOD was improved to 0.01 ng/mL. However, the new method provided a higher S/N ratio. Thus, we determined to use this new method to analyze the fluorescence intensity in the following experiments.

### SFSI-enhanced immunoassay for a simulated saliva sample using an ultra-thin immuno-wall

**Fig. 6:**
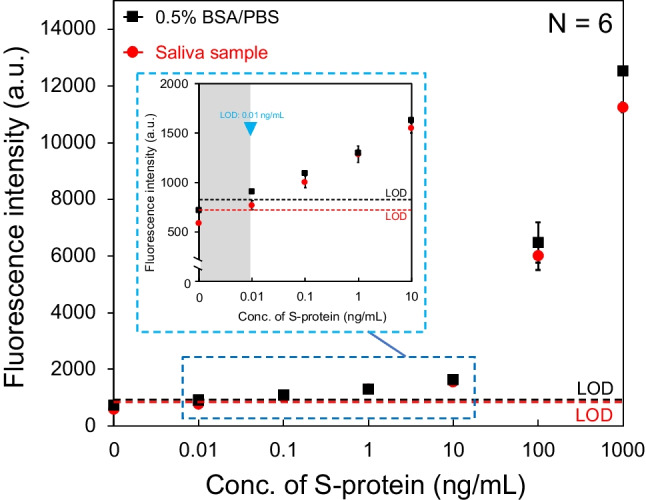
Calibration curves for the S-protein in 0.5% BSA/PBS buffer and saliva sample using the ultra-thin immuno-wall with the new analytical method

The proof-of-concept experiment of SFSI-enhanced immunoassay of S-protein in the simulated saliva sample was designed and conducted. To simulate the physical properties of the real clinical saliva swab with similar components, a saliva sample was obtained from a volunteer engaged in this research (RT-PCR negative), by using a commercial saliva swab kit, following the manuals. The operation time for the collection of one saliva sample was about 5 min. Then, the S-protein was diluted to the simulated saliva sample to afford solutions with concentrations of 1000, 100, 10, 1, 0.1, 0.01, and 0 ng/mL. A plot of the fluorescence signal vs. S-protein concentration is shown in Fig. 6. The LODs for the S-protein in 0.5% BSA/PBS buffer and the simulated saliva sample were 0.01 ng/mL, which is a 1000-fold increase over the conventional sandwich fluorescence immunoassay and a 10-fold increase over the commercialized ELISA [36, 37]. The detection time was 30 min, which was the same as the rapid antigen test, whereas it is 1/6 that of ELISA and 1/20 that of RT-PCR. The sample consumption was only 0.6 μL. This LOD was sufficient for the detection of S-protein in the early infection phases.

We compared the performance of our immunoassay platform with other benchmark detection methods as shown in Table 2. Compared to viral RNA and N-protein that are used in RT-PCR and rapid antigen tests, the S-protein is a surface protein; therefore, there is no need for extraction, which significantly shortens the operation time of the immunoassay. Furthermore, the S-protein is more stable than viral RNA, which is a crucial factor related to sensitivity and stability [33]. Overall, our immunoassay platform exhibited a short detection time and a high sensitivity.

**Table 2:** Benchmark comparison of detection methods for the SARS-CoV-2 virus

	This study	ELISA [34]	Rapid antigen test [21]	RT-PCR [23]
Biomarker	Viral proteins (S-protein)	Viral proteins/antibodies	Viral proteins (N-protein)	Viral RNA
Stability of biomarker	+++	+++	+++	++
Sensitivity	0.01 ng/mL	0.1 ng/mL	5-10 ng/mL	100–1000 copies/mL (0.01–0.1 ng/mL)
Detection time	30 min	3-4 h	30 min	24-48 h

## Conclusions

In this study, we developed a rapid and highly sensitive immunoassay platform using an ultra-thin immuno-wall combined with an easy-to-perform SFSI method. The SFSI method afforded an enhanced fluorescence signal for samples with low concentrations, and the ultra-thin immuno-wall served to reduce the nonspecific binding and increase the S/N ratio for the immunoassay. Our immunoassay platform exhibited a high sensitivity of 0.01 ng/mL with a rapid detection time of 30 min for the S-protein of the SARS-CoV-2 virus. This sensitivity matches the loads of the spike protein in the early phases of infection. Hence, our immunoassay platform can be a potential solution to address the urgent demand for time-sensitive detection such as COVID-19 and other infectious diseases. Furthermore, in this study, to fulfill the clinical requirements for rapid detection, fluorescence-labeled antibodies were employed four times to enhance the fluorescence signal, and this improved sensitivity reached the level needed for early-phase detection of the SARS-CoV-2 virus. In the future, by increasing the incubation cycles of fluorescence-labeled antibodies, our immunoassay platform will make it possible to detect a wide range of biomarkers at lower sample loads. Furthermore, since our SFSI-enhanced immunoassay involves easy-to-perform and repeated incubation of the fluorescence-labeled antibodies to enhance the signal intensity, it has the potential to be integrated into an automatic immunoassay platform for clinical applications such as early disease diagnosis, point-of-care testing, and precision medicine.

## Supplementary Information

Below is the link to the electronic supplementary material.

Supplementary file1 (PDF 1274 KB)

## Data Availability

All data generated during this study are included in this published article and its supplementary information files. Raw experimental data and additional methodological details are available from the corresponding author upon reasonable request.

## Declarations

### Biosafety considerations

All experiments involving biological samples were conducted in compliance with institutional biosafety guidelines and appropriate laboratory safety protocols. The simulated saliva samples were confirmed to be COVID-19 negative by RT-PCR prior to use in this research.
