# Supplementary material for: Rapid and highly sensitive immunoassay using an ultra-thin immuno-wall microfluidic device with a sequential fluorescence signal increment method
Source: Anal Bioanal Chem. 2025 May 28;417(17):3935–44. doi: 10.1007/s00216-025-05916-x (PMC12227451; doi:10.1007/s00216-025-05916-x)
Supplement: Supplementary file 1 — Supplementary file1 (PDF 1274 KB) [file 216_2025_5916_MOESM1_ESM.pdf]

Rapid and highly sensitive immunoassay using an ultra-thin immuno-wall microfluidic device with a sequential fluorescence signal increment method

Xiang Zhou[1], Toshihiro Kasama[1], Ryo Miyake[1]

1. Department of Bioengineering, Graduate School of Engineering, The University of Tokyo

Supplementary Material I

A B C D

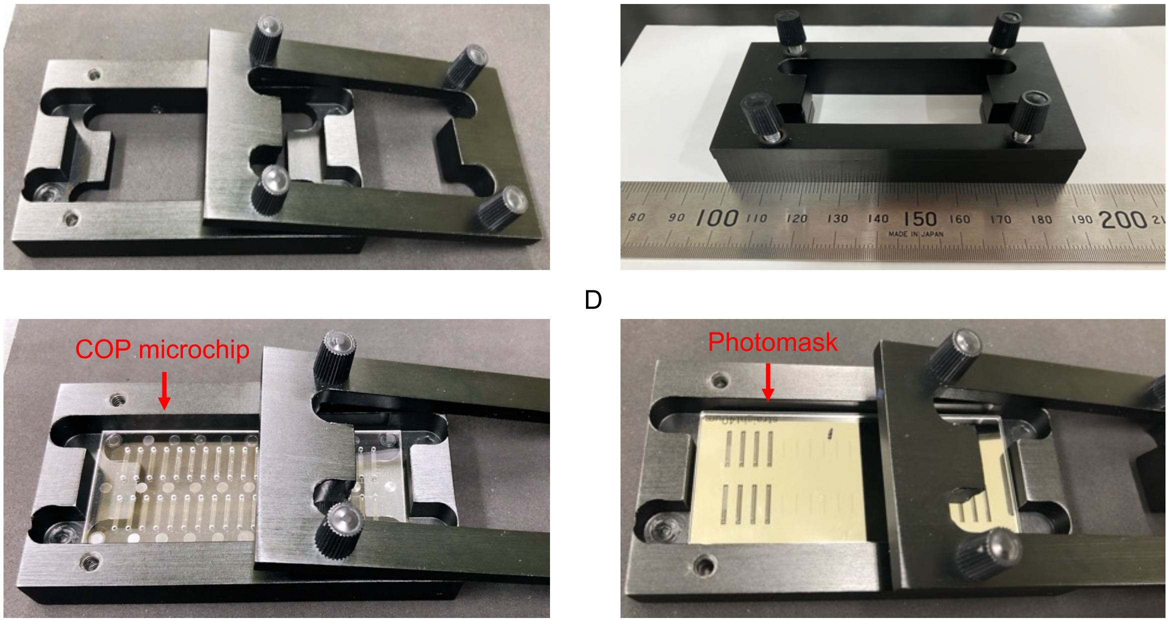

Fig. S1 (A) Photograph of alignment holder. (B) Alignment holder with a scale (smallest division: 1 mm). (C) COP chip inside alignment holder. (D) COP chip with aligned photomask inside alignment holder.

Supplementary Material II

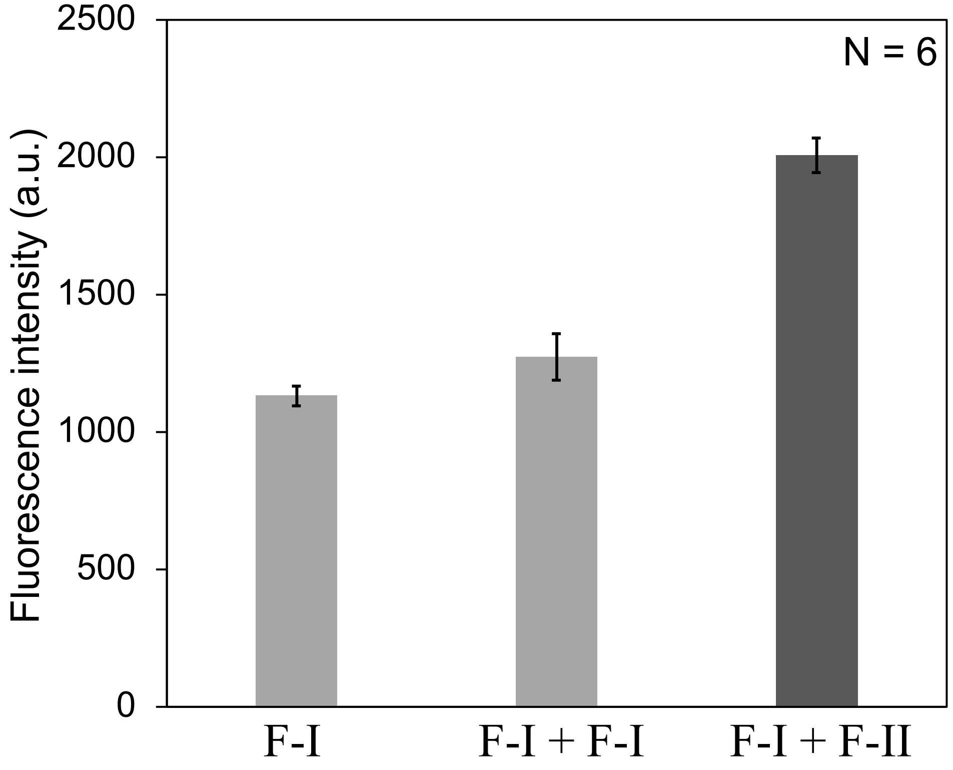

Fig. S2 Bar graph of the confirmation of the formation of multi-layer immune complex.

(S-protein: 100 ng/mL)

Supplementary Material III

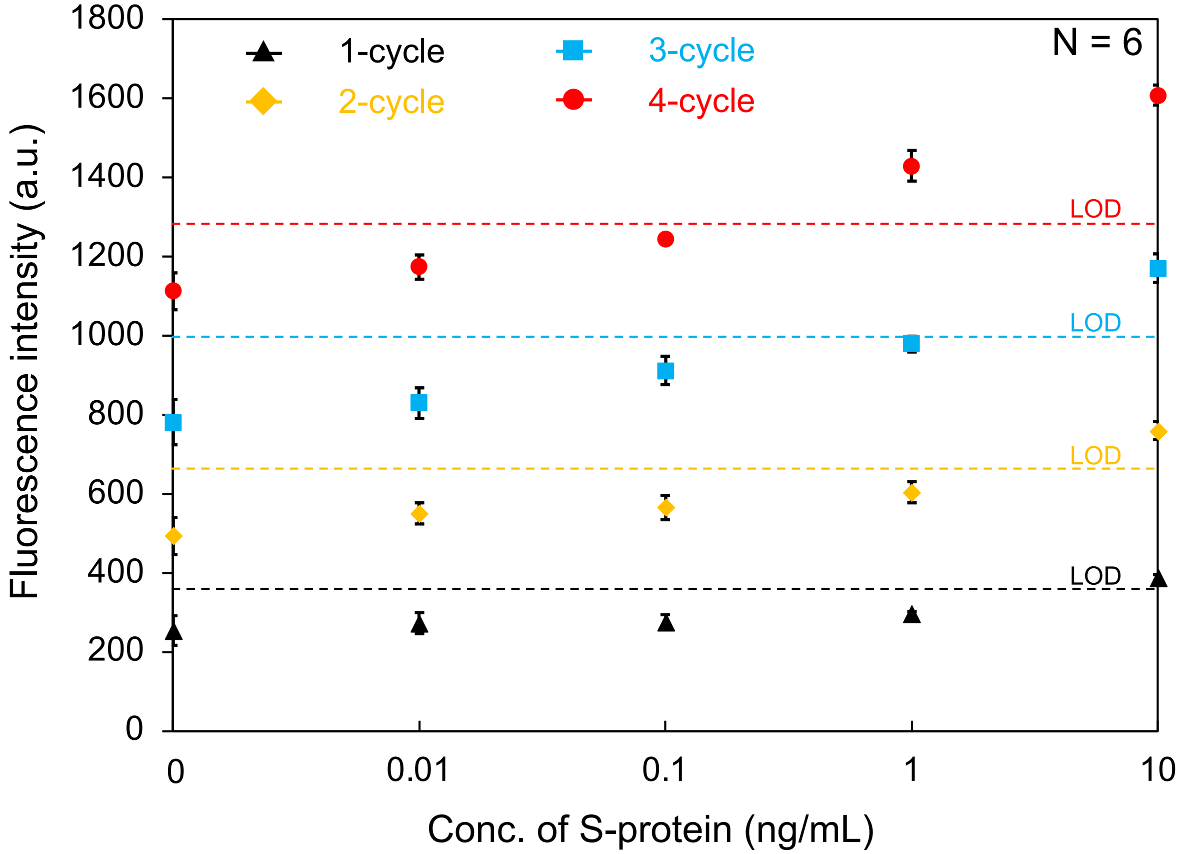

Fig. S3 Plot of fluorescence intensity vs concentration of S-protein in 0.5% BSA/PBS buffer with different incubation cycles of fluorescence-labeled antibody using conventional immuno-wall. (1-cycle: F-I → 2-cycle: F-II → 3-cycle : F-I → 4-cycle : F-II). The value of fluorescence intensity was plotted as the mean ± 3SD (N = 6). The dotted line represents the LOD which is 3 times of SDs above the negative control (0 ng/mL).

Supplementary Material IV

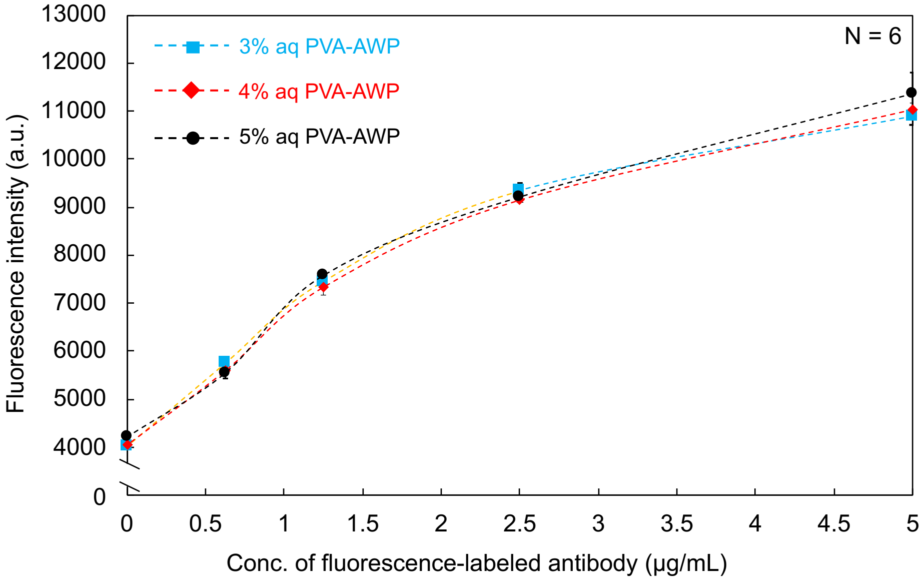

Fig. S4 Plot of fluorescence intensity vs concentration of fluorescence-labeled antibody (5, 2.5, 1.25, 0.625 μg/mL) with different concentrations of PVA-AWP. In this experiment, 50 μg/mL immobilized antibody was used for immobilization. To estimate the immobilization ability of the immuno-wall with different cross-linking densities, DyLight 650-conjugated rabbit anti-human polyclonal IgG antibody diluted as 5, 2.5, 1.25, and 0.625 μg/mL was used to react with immobilized antibodies.

Supplementary Material V

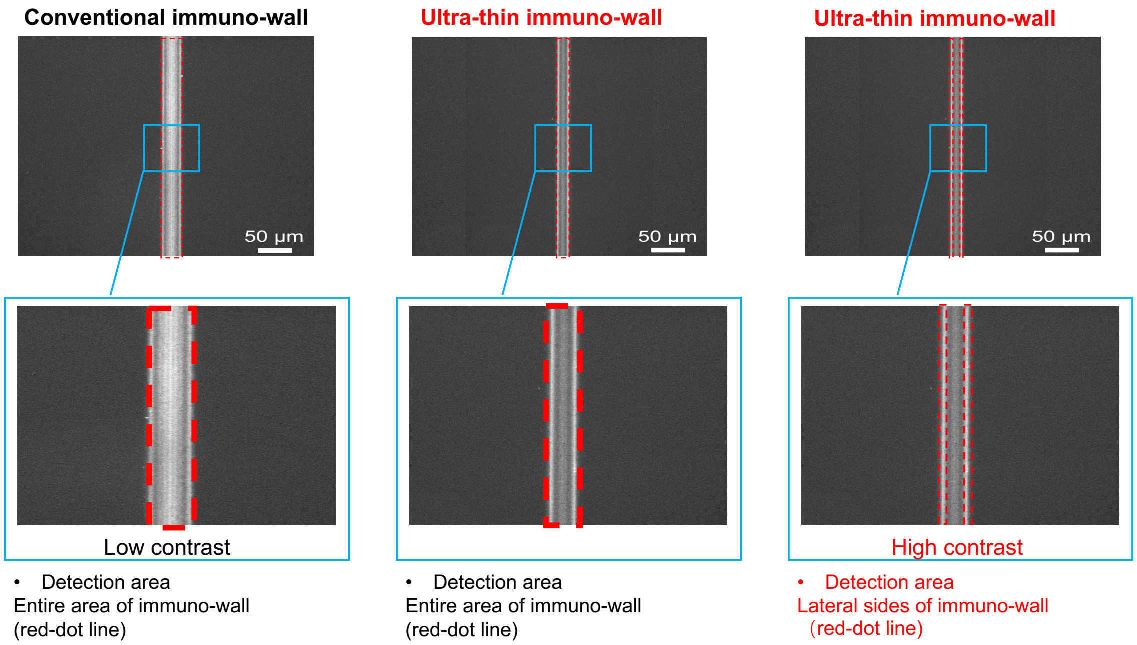

$$Fluorescence\ intensity\ of\ each\ image = \frac{\sum Value\ of\ each\ pixel\ in\ selected\ area}{number\ of\ pixel}$$

❖ *The mean value of the fluorescence intensity increases when the lateral-side area is selected as the detection area, due to the decreased number of pixel from the middle area of immuno-wall.*

Fig. S5 Fluorescence intensity analysis based on different detection areas.
